# Augmented thrombin formation is related to circulating levels of extracellular vesicles exposing tissue factor and citrullinated histone-3 in anti-neutrophil cytoplasmic antibody-associated vasculitides

**DOI:** 10.3389/fmed.2023.1240325

**Published:** 2023-10-17

**Authors:** Asta Dogg Jonasdottir, Milena Manojlovic, Jelena Vojinovic, Annica Nordin, Annette Bruchfeld, Iva Gunnarsson, Fariborz Mobarrez, Aleksandra Antovic

**Affiliations:** ^1^Department of Clinical Science, Intervention and Technology, Division of Renal Medicine, Karolinska Institutet, Stockholm, Sweden; ^2^Division of Nephrology, Department of Medicine, Landspitali – The National University Hospital, Reykjavik, Iceland; ^3^Department of Pediatrics, Medical Faculty, University of Niš, Niš, Serbia; ^4^Department of Medicine, Division of Rheumatology, Karolinska Institutet, Stockholm, Sweden; ^5^Department of Health, Medicine and Caring Sciences, Linköping University, Linköping, Sweden; ^6^Rheumatology, Karolinska University Hospital Stockholm, Stockholm, Sweden; ^7^Department of Medical Sciences, Uppsala University, Uppsala, Sweden

**Keywords:** anti-neutrophil cytoplasmic antibody (ANCA)-associated vasculitis, extracellular vesicles, thrombin generation, NETs, tissue factor

## Abstract

**Objectives:**

To study circulating myeloperoxidase (MPO)-positive extracellular vesicles (MPO^+^EVs) exposing citrullinated histone-3 (H3Cit), tissue factor (TF), and plasminogen (Plg) in association to thrombin generation in patients with anti-neutrophil cytoplasm antibody (ANCA)-associated vasculitis (AAV).

**Methods:**

We have involved well-characterized patients with AAV together with population-based controls. Flow cytometry was used to assess the levels of MPO^+^EVs in citrated plasma. MPO^+^EVs were phenotyped by anti-MPO-antibodies together with anti-CD142 (anti-TF), anti-H3Cit, and anti-Plg antibodies. A modified Calibrated Automated Thrombogram (CAT) assay was utilized to measure thrombin generation in plasma initiated by EVs-enriched pellets. The activity of AAV was evaluated with the Birmingham Vasculitis Activity Score (BVAS).

**Results:**

This study comprised 46 AAV patients, 23 in the active stage of the disease and 23 in remission, as well as 23 age- and sex matched population-based controls. Augmented levels of all investigated MPO^+^ EVs were found in active AAV patients in comparison to the subgroup of patients in remission and controls. Thrombin generation, measured by endogenous thrombin potential (ETP) and peak of thrombin formation, was higher in plasma when triggered by EVs-enriched pellet from AAV patients. ETP and peak were associated with the levels of MPO^+^TF^+^ and MPO^+^H3Cit^+^ EVs. Additionally, MPO^+^TF^+^ EVs correlated with the disease activity evaluated with BVAS.

**Conclusion:**

Augmented thrombin generation is found in AAV patients regardless of disease activity and is associated with higher exposure of TF and H3Cit on MPO^+^EVs. This may contribute to the increased risk of thrombosis seen in AAV patients.

## Introduction

Antineutrophil cytoplasmic antibody (ANCA) associated vasculitis (AAV) is a group of necrotizing vasculitides predominantly affecting small- to medium-sized blood vessels, in the presence of proteinase-3 (PR3) and myeloperoxidase (MPO) ANCAs (1). AAVs have a wide range of organ manifestations, which can lead to vital organ failure and carry a high mortality risk, if left untreated (2).

Although the prognosis for AAV patients has improved substantially after introduction of modern immunosuppressive treatment, an amplification of treatment- and disease-related complications remains a challenge. AAV is associated with a considerably increased risk for venous thromboembolism (VTE) in comparison to the general population and even to other auto immune inflammatory diseases such as systemic lupus erythematosus and rheumatoid arthritis (3, 4). The risk is the highest in patients with active disease but is even increased during remission (5–7).

The pathogenesis of AAV is not fully understood, but current findings point towards essential role of ANCA-induced activation of neutrophils (8, 9). Following priming of neutrophils and activation by ANCA, inflammatory process arises, including neutrophil degranulation and release of neutrophil extracellular traps (NETs) (10). NETs are web-like structures containing DNA and histones that have proinflammatory properties and contribute to injury of endothelial cells (11).

During NETosis neutrophils release extracellular vesicles (EVs), submicron membranous vesicles that are shed from the cell surface, containing membrane markers of the parental cells and may exert pro-inflammatory and pro-coagulant effects (12). Neutrophil derived EVs (NEVs) containing MPO have been shown to contribute to damage of vascular endothelial cells (13, 14). MPO primarily originates from neutrophil granules, but may also be found in monocytes, though at much lower concentrations (15). Therefore, the majority of the MPO^+^EVs can be anticipated to derive from neutrophils.

After activation of neutrophils and in the process leading up to NETosis, histone-3 is citrullinated by peptidyl arginine deiminase 4 (PAD4) in neutrophils. Citrullinated Histone-3 (H3Cit) is released in NETs and serves as a specific marker for NETs (16). H3Cit has recently been shown to be exposed on EVs in human model of endotoxemia (17). Previous studies of H3Cit exposure on EVs in systemic inflammatory diseases are lacking, while there are reports of elevated tissue factor (TF) expression on circulating NEVs in patients with AAV (18).

Plasminogen (Plg) is a crucial proenzyme of the fibrinolytic system, one of the major regulating systems in hemostatic process (19). Anti-plasminogen antibodies were verified in a subgroup of patients with AAV and were associated with disease activity, VTE and occurrence of glomerular fibrinoid necrosis, cellular crescents, as well as kidney function impairment (20–23).

This study was aimed to investigate the presence of H3Cit, TF and Plg on circulating MPO^+^EVs in relation to thrombin generation triggered by the EVs-enriched pellet extracted from plasma of patients with AAV. Thereby we aimed to investigate mechanisms contributing to a prothrombotic state in these patients.

## Materials and methods

### Patients and methods

This cohort of AAV patients included in this study was previously described in recently published studies (24, 25). Briefly, 46 patients with AAV and 23 population-based, age- and sex-matched controls were investigated. The diagnosis of granulomatosis with polyangiitis (GPA) or microscopic polyangiitis (MPA) was made either at the Department of Nephrology or Rheumatology at Karolinska University Hospital. ANCA-positivity (ever) was required for the inclusion in the study. The vasculitis disease activity was assessed by the Birmingham Vasculitis Activity Score (BVAS, version 2003), in agreement to the European League Against Rheumatism (EULAR) recommendations (26). Definition of remission was BVAS 0. Glucocorticoid (GC) cumulative dose was calculated in prednisolone-equivalent milligrams (mg). The presence of significant haematuria in urine samples and/or elevated plasma creatinine levels indicated kidney involvement in the course of AAV. Kidney biopsy was performed in the majority of patients revealing pathological findings of pauci-immune vasculitis. Control samples were obtained from population based age- and sex-matched subjects (27).

The Regional Ethical Review Board in Stockholm and the Swedish Ethical Review Authority, approved the study protocol and informed consent was obtained from all included subjects.

### Blood sampling

Blood sampling and collection of peripheral venous blood was performed according to routine clinical and laboratory practice. We used Vacutainer tubes (Becton Dickinson) with clot activator or trisodium citrate (0.129 mol/L, pH 7.4). The collected blood samples were transferred from the clinical department to the laboratory within 1 h at room temperature and the samples were stored in the upright position. The collected venous blood was centrifuged at 2000 × g for 20 min at room temperature, aliquoted and stored at −70°C.

### Flow-cytometry assessment of EVs

To proceed to further analysis, PPP was first centrifuged at 2000 g for 20 min and thereafter at 13000 g for 2 min at room temperature. As previously described (24, 25), the supernatant was incubated with monoclonal antibodies in order to phenotype EVs with antibodies against MPO, conjugated with PE (anti-MPO-PE, Beckman Coulter, Brea, CA, United States), tissue factor (CD142D, NJ, United States), citrullinated histone-3 (anti-H3Cit, Abcam, Cambridge, United Kingdom), and plasminogen (Abcam, Cambridge, United Kingdom). The samples were further fixed (Cellfix, BD, NJ, United States) in order to be evaluated by flow cytometry on a Beckman Gallios instrument (Beckman Coulter, Brea, CA, United States). The EVs gate was determined using Megamix beads (0.3–1.0 μm, BioCytex, Marseille). EVs were defined as particles <1 μm in diameter, according to MISEV guidelines defnition of a medium/large EVs (28). The measured levels of EVs are presented as EVs/μL plasma. The intra- and inter-assay coefficients of variation for MPO^+^EVs measurement were <9%, respectively.

### Activity assay

To further analyse EVs and their procoagulant effect on thrombin generation, we used adjusted laboratory assay Calibrated Automated Thrombogram (CAT) (29). Commercial Thermo Scientific Flouroskan Ascent (Thermo Scientific) instrument was utilized together with transparent u-bottom 96-well plates. Briefly, EVs-enriched pellet originated from PPP after centrifugation at 2000 g for 20 min (500 μL plasma) and thereafter the supernatant was moved to a new tube and centrifuged at 20000 g for 45 min, at room temperature (RT) as described previously (30). Following the first high-speed centrifugation, the supernatant was removed, leaving 50 μL of a EV-enriched suspension which was further diluted with 450 μL of phosphate-buffered saline (PBS Buffer, pH 7.6) and centrifuged again for 45 min at 20,000 g at RT. Twenty microliters of pellet were then used as a trigger of thrombin formation, instead of TF and phospholipids originally utilized in the CAT assay. EVs-enriched pellet was added to EV-poor supernatant obtained from centrifuged normal pool plasma (NPP) from healthy controls (20,000 g for 45 min at RT). The EV-poor plasma was also used for control measurements (i.e., plasma containing no EVs and no trigger). The pellet from all patients and all control samples was used. The period of time for measurement of thrombin generation was 60 min at 37°C, as in the original CAT assay. Thrombin generation curve (Thrombogram) was generated by measuring fluorescence that was detected each 20 s during this period and thrombin curve (Thrombogram) was created. Different parameters were calculated from Thrombogram by the commercial analyzing software (Thrombinoscope).

**Figure 1:**
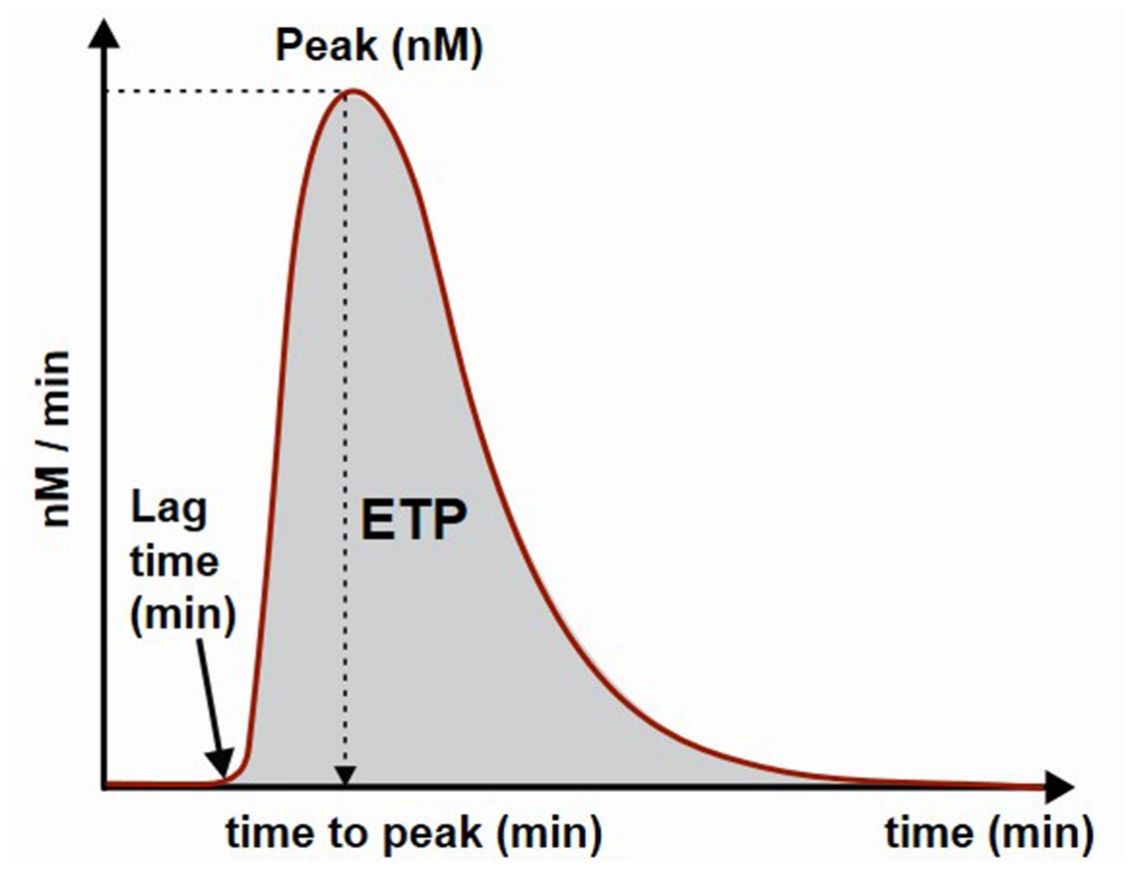
Schematic presentation of a thrombin generation curve and parameters. ETP, endogenous thrombin potential.

The diagram of thrombin formation is presented in Figure 1. The main parameter of thrombin generation is endogenous thrombin potential (ETP) and is calculated as area under the thrombin curve. Additional parameters are: the lag time which is the time needed to start thrombin generation; the peak of thrombin generation representing maximal concentration of thrombin formed during the investigated time period and the time to peak which is the time needed to achieve maximal thrombin generation.

### Routine laboratory analyses

ANCAs were detected by standard Enzyme-Linked Immunosorbent Assay methods, either a direct ELISA (Euro diagnostic) or multiplex (BIO-RAD, BioPlex TM 2200) according to clinical routine at Karolinska University Hospital Immunology Department.

Standard laboratory analyses were performed at the Clinical Chemistry Department, Karolinska University Hospital, to assess serum C-reactive protein (CRP), erythrocyte sedimentation rate (ESR) and plasma creatinine levels. The estimated glomerular filtration rate (eGFR) was calculated using the Chronic Kidney Disease Epidemiology Collaboration (CKD-EPI) equation (31).

### Statistical analysis

Descriptive statistics were used for the presentation of patient characteristics. For continuous variables, means and standard deviations or medians with 25th–75th percentiles were used, whereas categorical variables were presented as percentages. Distribution of data was checked by the Shapiro–Wilk test. Independent samples t tests (parametric) and Wilcoxon rank sum tests (non-parametric) were used to assess the difference in estimated variables between the groups. For comparison of more than two groups the Kruskal–Wallis analysis of variance (ANOVA) was used. The correlation between variables was calculated using the Spearman correlation analysis. A *p*-value <0.05 was regarded as statistically significant. Data were analysed using JMP software (version 14; SAS, Cary, NC, United States) and GraphPad Prism (version 9; GraphPad Software, La Jolla, CA, United States).

## Results

### General patient data

Forty-six AAV patients were involved in the study, 23 in the active stage of the disease and 23 in remission. Table 1 comprises general characteristics of the patients like age, sex, AAV disease phenotype, ANCA type at diagnosis, kidney function and disease activity score (BVAS). Twenty-three population-based controls were age- and sex-matched to the patients. All active patients had recent onset of disease (*n* = 22) or a flare (*n* = 1) prior to the inclusion in the study, where the median time from diagnosis to inclusion and blood sampling was 4 days (0 to 177 days). Median disease duration in inactive patients was 5.3 (1.2–12) years. Kidney biopsy was performed in 13 patients with active disease, while kidney involvement was diagnosed in one patient only based on elevated plasma creatinine level and haematuria. Cardiovascular and VTE events were not previously diagnosed among patients included in the study. At the time of blood sampling, investigated subjects were not treated with anticoagulant treatment and were not subjected to plasma exchange.

**Table 1:** General characteristics of the patients and controls.

	Total AAV (*n* = 46)	Controls (*n* = 23)	*P*	Active AAV (*n* = 23)	Inactive AAV (*n* = 23)	*P*
Sex, F/M	21/25	11/12	1.00	10/13	11/12	1.00
Age, years	65 (24–81)	64 (49–81)	0.27	63 (24–81)	65 (26–81)	0.55
MPA	23 (50)			13 (56.5)	10 (43.5)	0.56
GPA	23 (50)			10 (43.5)	13 (56.5)	0.56
MPO-ANCA	27 (58.7)			14 (60.9)*	13 (54.2)*	0.77
PR3-ANCA	21 (45.7)			10 (43.5)*	11 (47.8)*	0.77
BVAS	1.5 (0–14)			14.0 (7–19)	0 (0)	N/A
Creatinine, μmol/L	99.0 (78.3–148.5)	74.5 (66.8–84.0)	**<0.001**	103.0 (79–198)	98.0 (76–124)	0.45
eGFR, mL/min/1,73 m^2^	64.5 (45.3–85.3)	88.5 (78.8–97.3)	**0.001**	65.0 (27.0–87.0)	64.0 (49.0–79.0)	0.78
CRP, mg/L	6.0 (2.0–12.5)			10.0 (6.0–17.0)	2.0 (1–9)	**0.002**
ESR, mm/h	21.0 (10.0–42.0)			39.5 (13.75–59.5)	15.0 (9.5–29.0)	**0.03**
Kidney involvement	34 (73.9)			14 (60.9)	19 (82.6)	0.19
Treatment at sampling						
GCs	37 (80.4)			22 (95.7)	15 (65.2)	**0.02**
Prednisolone dose, mg/day	13.8 (4.4–66)			60.0 (40–75.0)	5.0 (0–10.0)	**<0.001**
Methotrexate	7 (15.2)			3 (13.0)	4 (17.4)	1.00
Azathioprine	7 (15.2)			1 (4.3)	6 (26.1)	0.10
Mycophenolate mofetil	6 (13)			1 (4.3)	5 (21.7)	0.37
Cyclophosphamide	5 (10.9)			5 (21.7)	0 (0)	0.02

Data is shown as *n* (%) or median (25th–75th percentiles). AAV, antineutrophil cytoplasmic antibody (ANCA)-associated vasculitis; F, female; M, male; GPA, granulomatosis with polyangiitis; MPA, microscopic polyangiitis; PR3, proteinase 3; MPO, myeloperoxidase; BVAS, Birmingham vasculitis activity score; eGFR, estimated glomerular filtration rate; CRP, C reactive protein; ESR, erythrocyte sedimentation rate; GCs, glucocorticoids. ^*^One patient in each subgroup (active and inactive group) was positive for PR3 and MPO-ANCA. Statistically significant *p* values (<0.05) are shown in bold.

Serum CRP and ESR levels were significantly higher in the active AAV group (*P* 0.002 and *P* 0.03, respectively) compared to patients in remission (Table 1). Patients with active AAV were treated with significantly higher median GC dose compared to inactive patients (*p* < 0.001). The median cumulative dose that the active AAV patients had received at baseline was 625 mg (range 0 to 3,825 mg). Cyclophosphamide treatment was administrated in five of the active AAV patients prior to sampling, however none of the patients had received more than one dose.

### Expression of EVs in different subgroups

**Figure 2:**
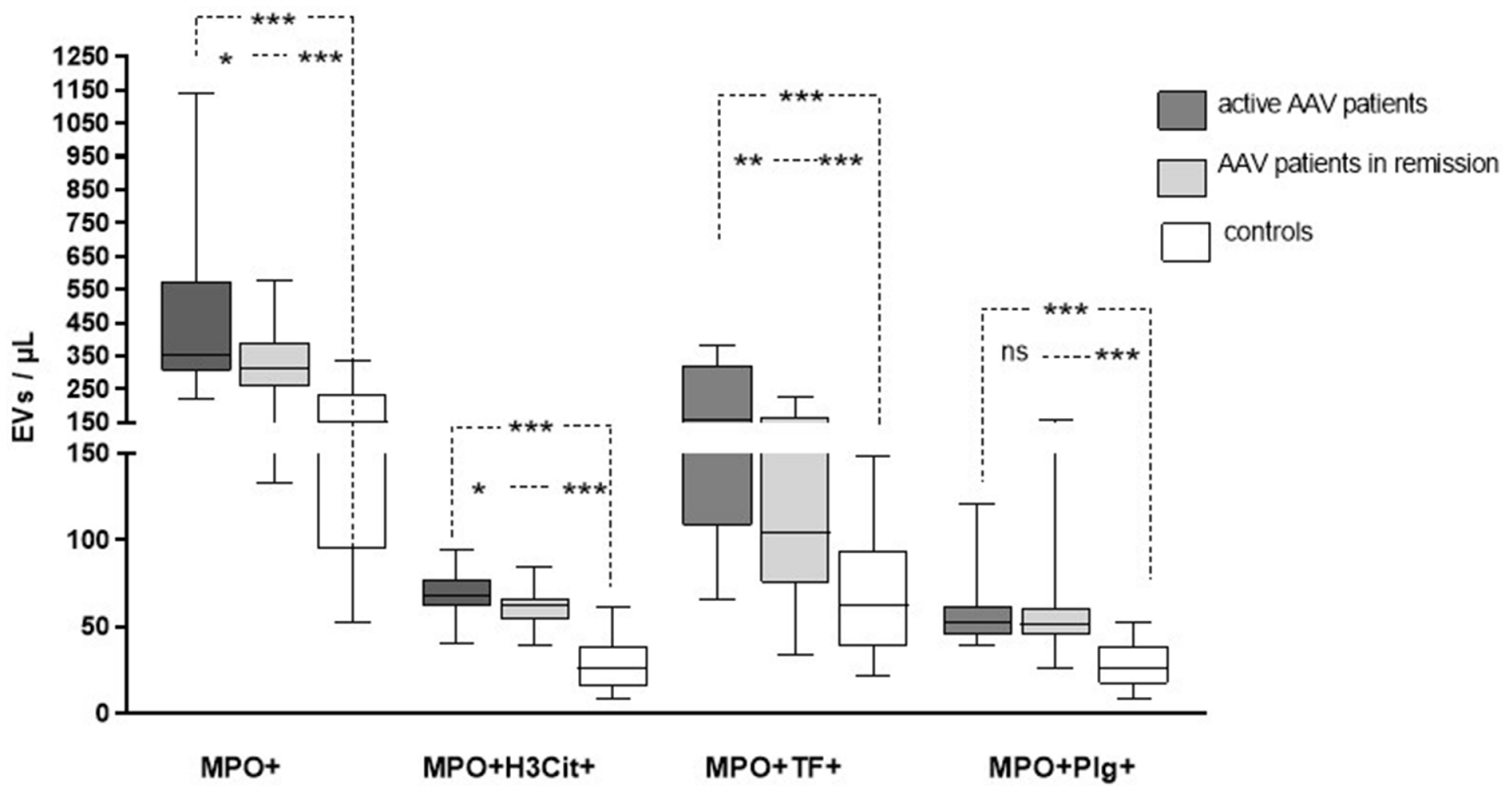
The levels of extracellular vesicles (EVs) exposing myeloperoxidase (MPO), citrullinated histone-3 (H3Cit), tissue factor (TF), and plasminogen in patients with active and inactive ANCA-associated vasculitis (AAV) compared to control subjects.

The levels of circulating MPO^+^EVs detected in plasma from patients with AAV were increased compared to the levels found in controls, as well as MPO^+^EVs expressing TF (MPO^+^TF^+^ EVs), H3Cit (MPO^+^H3Cit^+^ EVs), and Plg (MPO^+^Plg^+^ EVs) as shown in Table 2 and Figure 2. Patients with active AAV had higher levels of MPO^+^TF^+^ EVs and MPO^+^H3Cit^+^ EVs compared to inactive patients, while there was no difference in levels of MPO^+^Plg^+^ EVs levels (Table 2). The levels of investigated EVs were not affected by kidney involvement.

**Table 2:** Levels of MPO^+^EVs and activity assay variables in ANCA-associated vasculitis (AAV) patients and controls.

	Total AAV (*n* = 46)	Controls (*n* = 23)	*P*	Active AAV (*n* = 23)	Inactive AAV (*n* = 23)	*P*
Extracellular vesicles (EVs/μL)						
MPO^+^	330.8 (274.0–427.9)	154.8 (94.8–236.0)	**<0.001**	353.0 (305.6–574.0)	311.4 (255.6–387.6)	**0.04**
MPO^+^TF^+^	122.2 (93.8–179.1)	63.0 (38.4–93.6)	**<0.001**	159.0 (108.6–322.0)	104.4 (75.6–165.0)	**0.02**
MPO^+^H3Cit^+^	63.8 (55.7–73.5)	26.4 (16.2–39.0)	**<0.001**	67.8 (61.8–77.0)	62.0 (54.0–66.0)	**0.03**
MPO^+^Plg^+^	51.8 (45.9–61.4)	26.4 (17.4–39.0)	**<0.001**	52.0 (46.0–61.8)	51.6 (45.6–61.2)	0.90
Activity assay						
Lag time (min)	17.3 (14.5–19.8)	20.1 (18.9–24,1)	**<0.001**	17.2 (15.7–19.7)	17.0 (13.7–20.4)	0.55
ETP (nM/min)	1269.0 (873.2–1693.6)	787.5 (459.0–829.5)	**<0.001**	1501.5 (946.8–1777.5)	1191.6 (797.5–1425.6)	0.28
Peak (nM)	76.8 (54.8–113.3)	48.2 (32.0–54.5)S	**<0.001**	100.3 (55.4–128.8)	67.2 (53.2–92.3)	0.20
ttPeak (min)	22.4 (20.2–25.4)	25.7 (24.6–29.7)	**<0.001**	22.4 (21.1–25.1)	21.8 (19.7–26.6)	0.58

Data is shown as median (25th to 75th percentile). MPO, myeloperoxidase; TF, tissue factor; H3Cit, Citrullinated Histone-3; Plg, Plasminogen; Lag time, time to start of thrombin generation; ETP, Endogenous thrombin potential, total amount of thrombin generated over time; Peak, the maximal concentration of thrombin generated; ttPeak, time to peak, the time to maximal thrombin generation. Statistically significant *p* values (<0.05) are shown in bold.

### Associations between clinical and laboratory variables with investigated parameters

There was a correlation between age and levels of MPO^+^EVs exposing TF and H3Cit (*ρ* = 0.59, *P* 0.003 and *ρ* = 0.46, *P* 0.03, respectively) in the subgroup of AAV patients with active disease, while a negative correlation was found between age and MPO^+^Plg^+^ EVs (*ρ*−0.42, 0.046) in these patients. Kidney function, measured by creatinine and eGFR, did not correlate with the levels of any of the investigated MPO^+^EVs.

For all AAV patients, the levels of MPO^+^TF^+^EVs correlated with disease activity determined by BVAS (*ρ* = 0.43, *P* 0.003). AAV activity measured by BVAS did not correlate with MPO^+^EVs expressing H3Cit or Plg. A correlation was found between the levels of circulating MPO^+^EVs uttering TF and H3Cit (*ρ* = 0.48, *p* < 0.001).

A correlation between the GC dose and the investigated markers was not found.

### Thrombin generation variables

Using a modified CAT assay we explored the ability of EVs to initiate thrombin formation in plasma. When EVs extracted from patients with AAV were used to trigger thrombin formation in NPP, we observed a significantly shorter lag time and time to peak of thrombin formation together with increased ETP and peak, compared to the same variables obtained when EVs from the control subjects were used (Table 2). However, there was no significant difference regarding thrombin generation when NPP was triggered with EVs either from patients with active or inactive disease.

For the whole AAV group, the peak correlated with the levels of MPO^+^TF^+^ EVs (*ρ* = 0.33, *P* 0.02) and MPO^+^H3Cit^+^ EVs (*ρ* = 0.44, *P* 0.003). A significant correlation was also seen between ETP and MPO^+^EVs exposing TF (*ρ* = 0.31, *P* 0.04) and H3Cit (*ρ* = 0.37, *P* 0.01). Further, a negative correlation was present between the levels of MPO^+^Plg^+^ EVs and the lag time (*ρ* = −0.41, *P* 0.005) and time to peak (*ρ* −0.33, *P* 0.03).

For active AAV patients a significant correlation was found concerning MPO^+^H3Cit^+^ EVs and time to peak (*ρ* = −0.45, *P* 0.03) and peak of thrombin formation (*ρ* = 0.44, *P* 0.04), while a negative correlation was confirmed between MPO^+^Plg^+^ EVs and the peak of thrombin formation (*ρ* = −0.42, *P* 0.047).

## Discussion

In this cross-sectional study in a cohort of AAV patients, we evaluated procoagulant markers expressed on MPO^+^EVs in relation to thrombin generation and disease activity. Samples of AAV patients were characterized by the presence of augmented levels of circulating MPO^+^EVs exposing H3Cit, TF and Plg compared to control samples. When tested during active phase of AAV, levels of MPO^+^EVs carrying TF and H3Cit were higher than in remission. Thrombin generation measured by ETP and peak was higher when triggered with EVs-enriched pellet from AAV samples compared to controls. Thrombin assembly in plasma correlated strongly with MPO^+^EVs expressing TF and H3Cit suggesting that these EVs may contribute to procoagulant state in AAV samples. Based on the present results, we speculate that MPO^+^EVs can have an impact on coagulation and thrombin generation, especially during the active phase of the disease.

TF is a main trigger of physiological coagulation process upon vessel wall damage and its intravascular exposure on the surface of blood cells. Circulating EVs exposing TF can bind and allocate lipids and proteins to activated platelets, thereby prompting thrombus formation (32, 33). Previously, Kambas et al. demonstrated TF expression on NETs and EVs derived from neutrophils upon stimulation by ANCAs obtained from samples during active stage of AAV. Moreover, there was a clear association between BVAS and the levels of neutrophil derived EVs expressing TF (18). Similar findings were reported by Huang *et al*, demonstrating that both PR3- and MPO-ANCA could stimulate the formation of NETs and neutrophil EVs. Additionally, they showed that NETs comprising TF influenced thrombin formation (34). The inflammatory burst and expression of TF may thus contribute to endothelial activation, amplification of vascular inflammation and hypercoagulability in AAV patients. Interestingly, there is also data demonstrating the occurrence of NETs in venous thrombi derived from a patient with AAV (35). Elevated EV TF activity have been found to be an indicator of VTE risk in AAV patients, even during remission (36). This is in line with our results, showing excess of MPO^+^EVs carrying TF and H3Cit not only in active disease, but also in patients in remission compared to healthy controls. Pronounced levels of these procoagulant EVs may play important role in the pathophysiology of thrombus formation in AAV. Although the thrombotic risk is highest in active disease, especially in patients with kidney involvement, it is still increased in remission (7).

We demonstrate an association between the levels of MPO^+^EVs exposing TF and H3Cit with variables of thrombin assembly in samples from AAV patients, although we must consider a potential role of EVs of other cellular origin on the thrombin formation in the present experimental setting. Notably, we have not investigated levels of EVs deriving from other cell types in the present study.

The thrombin generation curve (thrombogram) is a widely used tool in coagulation research, reflecting the procoagulant state in different groups of patients (37). In the current study we used adjusted laboratory assay to evaluate the effect of EVs-enriched pellet on thrombin formation. Instead of the addition of TF and phospholipids for initiation of thrombin generation, as described in the original CAT method, we here assess thrombin formation triggered by EVs-enriched pellet gained from plasma samples of AAV patients and control subjects. Thereby, we assessed the procoagulant properties of total amount of EVs extracted from investigated plasma samples. Similar modified assays have previously been described in studies of EVs in patients with recurrent thrombotic events and children with vasculitis (38, 39). The findings presented in this study postulate that functional procoagulant features of EVs at least partly depend on MPO^+^EVs carrying TF and H3Cit.

The role of Plg, a key proenzyme of fibrinolysis (18), has previously been studied in AAV. Antibodies directed to Plg may play a role in the disease pathogenesis as suggested by Bautz et al. who reported the presence of antibodies to Plg in a subgroup of patients with AAV. The postponed conversion of Plg to plasmin *in vitro* was influenced by the presence of antibodies against Plg and the occurrence of anti-Plg antibodies was associated with VTE in AAV patients (21). Elevated levels of anti-Plg antibodies were associated to AAV activity (20, 40) and fibrinolysis inhibition *in vitro*, together with more severe histologic lesions in the kidney as well as impaired kidney function in AAV patients (40). Although we did not find an association between Plg exposed on MPO^+^EVs and AAV activity, the levels were higher in AAV patients than in controls. These findings may be connected with the role of Plg in inflammatory reaction in AAV. Alongside the role in fibrinolysis Plg, and its active form plasmin, is involved in the recruitment of immune cells, resolving inflammatory processes and in interaction with the components of the complement system (41, 42).

The strength of our study is the assessment of EVs in well-defined groups of AAV patients without ongoing anticoagulant treatment and thereby a more specific evaluation of the possible impact of disease activity on the expression of investigated EVs. Moreover, we have investigated functional activity of EVs using thrombin generation assay. The limitations of our study are the relatively low number of patients included and the cross-sectional design. Moreover, we were unable to extract particular EVs from the pellet and thereby we investigated combined effect of EVs of different cell origin on thrombin generation. Longitudinal follow-up studies and more sophisticated isolation of EVs for functional experiments may contribute to a better understanding of how changes in EV levels associate with the increased coagulability in AAV in active disease.

In conclusion, we found that levels of MPO^+^TF^+^EVs were associated with activity of AAV and together with MPO^+^H3Cit^+^ EVs affected thrombin formation in the samples from AAV patients. These findings may contribute to an enhanced insight of the hypercoagulability and vascular injury seen in patients with AAV.

## Data availability statement

The raw data supporting the conclusions of this article will be made available by the authors, without undue reservation.

## Ethics statement

The studies involving humans were approved by The Regional Ethical Review Board in Stockholm and the Swedish Ethical Review Authority. The studies were conducted in accordance with the local legislation and institutional requirements. The participants provided their written informed consent to participate in this study.

## Author contributions

AJ collected samples and reviewed patient charts. MM and FM performed the experiments. AJ and AA performed statistical analyses. AA and IG designed the study. AA, IG, and AB supervised the manuscript. All authors contributed to the article and approved the submitted version.
